# Resource Competition May Lead to Effective Treatment of Antibiotic Resistant Infections

**DOI:** 10.1371/journal.pone.0080775

**Published:** 2013-12-13

**Authors:** Antonio L. C. Gomes, James E. Galagan, Daniel Segrè

**Affiliations:** 1 Bioinformatics Program, Boston University, Boston, Massachusetts, United States of America; 2 Department of Biomedical Engineering, Boston University, Boston, Massachusetts, United States of America; 3 Broad Institute of MIT and Harvard, Cambridge, Massachusetts, United States of America; 4 Department of Biology, Boston University, Boston, Massachusetts, United States of America; University of Melbourne, Australia

## Abstract

Drug resistance is a common problem in the fight against infectious diseases. Recent studies have shown conditions (which we call antiR) that select against resistant strains. However, no specific drug administration strategies based on this property exist yet. Here, we mathematically compare growth of resistant versus sensitive strains under different treatments (no drugs, antibiotic, and antiR), and show how a precisely timed combination of treatments may help defeat resistant strains. Our analysis is based on a previously developed model of infection and immunity in which a costly plasmid confers antibiotic resistance. As expected, antibiotic treatment increases the frequency of the resistant strain, while the plasmid cost causes a reduction of resistance in the absence of antibiotic selection. Our analysis suggests that this reduction occurs under competition for limited resources. Based on this model, we estimate treatment schedules that would lead to a complete elimination of both sensitive and resistant strains. In particular, we derive an analytical expression for the rate of resistance loss, and hence for the time necessary to turn a resistant infection into sensitive (*t_clear_*). This time depends on the experimentally measurable rates of pathogen division, growth and plasmid loss. Finally, we estimated *t_clear_* for a specific case, using available empirical data, and found that resistance may be lost up to 15 times faster under antiR treatment when compared to a no treatment regime. This strategy may be particularly suitable to treat chronic infection. Finally, our analysis suggests that accounting explicitly for a resistance-decaying rate may drastically change predicted outcomes in host-population models.

## Introduction

Drug resistance is an important problem during infection treatment, particularly in intensive care units [1]. Cases of resistance have been described in infections caused by different types of pathogens, such as viruses, bacteria, fungi and protozoa [2]–[5] and the increasing incidence has made resistance a major public health issue [6]. This fact can be exemplified by, but it is not exclusive to, infections caused by the methicillin-resistant *Staphylococcus aureus* (MRSA), whose incidence rate has almost doubled (city of Atlanta) or tripled (city of Baltimore) in a period of three years, from 2002 to 2005 [6]. The relevance of those numbers is evident when compared to infectious diseases that are caused by other bacteria also common in the human respiratory tract and skin, such as *Streptococcus pneumonia* and *Haemophilus influenzae*. The number of MRSA infection cases was about twice and 30 times the numbers for *S. pneumonia* and by *H. influenza*, respectively, in the calendar year of 2005 and was associated with about 18000 deaths [6]. Also, MRSA is associated with over 20% of *S. aureus* infections in Europe [7]. This alarming situation highlights the need for alternatives to reduce the incidence of resistance. Two common potential strategies for this purpose are drug restriction and multiple-drug therapy. However more work is required to determine the potential effectiveness of these strategies in reducing or fighting drug resistance and to gain a quantitative understanding of their mechanisms, both at the single-host and the host-population level.

Drug restriction consists of suspending a given class of antibiotics for some period of time, while other classes of antibiotics are still available for treatment. It is based on the principle that resistance can decrease in the absence of a specific antibiotic treatment, due to the cost of resistance [8]–[11]. For example, an early clinical study at the host-population level reported a reduction in the proportion of Vancomycin-resistant bacteria from 47% to 15% using a Vancomycin restriction strategy [12].

A special case of restriction is drug cycling, in which restrictions to specific classes of drugs are alternated over some time interval. A review on the topic identified only four references rigorously investigating drug cycling [13]. Three of them reported cycling to be effective in reducing the incidence of resistance and one did not find any statistical significance. They also reported lack of standard procedures, which makes it hard to obtain a conclusive evaluation of policies. A parallel review was less stringent and observed that thirteen out of fourteen studies related to drug cycling reported positive results, such as decrease of either resistance, infection rate or mortality rate, while only one reported purely negative results [14]. Subsequent studies reported positive outcomes for drug cycling [15]–[21]. While one case reported a combination of positive and negative results [21], and another discussed drawbacks of this approach [17], all of them agreed that more research is needed to identify useful strategies to combat resistance.

Another option to deal with drug resistance is using multi-drug therapy. The properties of drug combinations have been studied for more than 100 years [22]–[24]. The nature of drug interactions can be classified in two main groups: synergistic and antagonistic. An interaction is classified as synergistic (antagonistic) if the combined use of two drugs increases (decreases) their activity, such as growth inhibition, relative to a null expectation based on individual drug effects [25]. In using drug combinations for therapeutic purposes, most research until recently has been focused on synergistic interactions [26]–[29]. Drug synergy reduces the amount of drug necessary to reach the same activity, consequently reducing costs and presumably toxicity to patients [26]. However, new studies have shown that synergistically interacting drugs tend to increase the emergence of drug resistance, indicating that it would be useful to pursue the potential role of antagonistic interactions in affecting the evolution of resistance [26], [30]–[32].

Resistant strains would not be so alarming if we were able to control them. In order to do so, one would have to find conditions (which we call antiR) in which sensitive strains are able to grow faster than resistant ones. Under these conditions, resistant strains would have a selective disadvantage and decrease in population size. The antiR conditions can be applied to reduce resistance, turning an infection susceptible to antibiotic treatment. The effectiveness of this strategy depends on a precise timing schedule for the application of antiR and antibiotic treatment.

The existence of antiR conditions have been demonstrated by experimental measurements [33], [34]. Chait and colleagues used suppressive interaction to favor the growth of a wild type, sensitive strain over the growth of a resistant one [33]. Suppressive interactions are a special case of antagonism, and occur when the combined effect of two drugs is weaker than the effect of each drug individually. A suppressive drug attenuates the effect of an active drug in the sensitive strain, but not in the one carrying the genes for resistance to the suppressive drug. Thus, it creates a condition that favors the growth of sensitive strains.

A second antiR mechanism is possible when resistance is acquired through the use of efflux pumps [34]. This machinery keeps the antibiotic outside the cell and is activated by the presence of the antibiotic. It is an expensive process, in which the antibiotic is actively transported against its gradient of concentration at expenditure of free energy. Modifications caused by chemical decay may cause an antibiotic to be no longer effective, while maintaining its capacity to activate the genes for resistance. Under these conditions, the modified antibiotic is not effective and the activation of the efflux pumps is not associated with any benefit for the bacteria. Thus, it only increases the cost of carrying and expressing the genes for resistance, favoring growth of sensitive strains.

In spite of the growing knowledge about antibiotic resistance, there is still not a standard way to control it. The use of drug combinations can lead to multi-resistant strains [35]–[38]. Specific strategies to turn antiR conditions into therapeutic plans have not been proposed yet. Drug restriction is not a well-established intervention, with limited studies available on the topic [14], [36]. Moreover, the implementation of drug restriction policies beyond a single hospital is challenging. In the case of cycling, lack of standard procedures and arbitrary definition of cycle duration are central issues [13], [14], [17], making strategies inconclusive. Mathematical models could help to improve strategies. However, most models [39]–[41] predict that antimicrobial cycling is not helpful in reducing resistance while most experimental investigations suggest benefits for cycling [14]. Such divergence encourages the search for the principles necessary to develop accurate models and highlights the importance of more experimental evidence.

**Figure 1:**
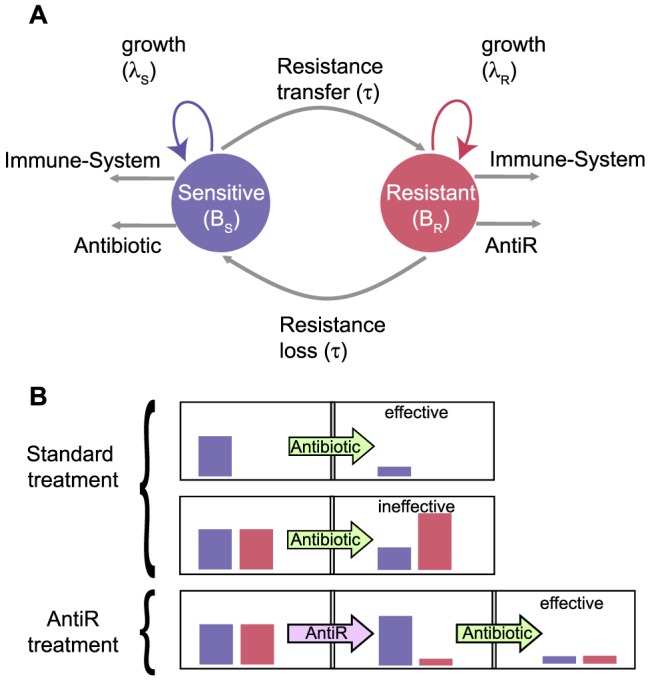
Illustration of the infection dynamics model and of a novel strategy to fight resistance. (*A*) Schematic representation of the main dynamical transitions based on the model from [42]. The arrows represent the possible fates of the populations of sensitive and resistant pathogen strains. Horizontal gene transfer (rate *τ*) and plasmid loss (rate *ρ*) are the mechanisms responsible for interconverting between sensitive and resistant strains. The use of an antibiotic can reduce the sensitive population, but is not effective against the resistant one. Conversely, the cost of carrying a plasmid causes a reduction of the resistant population in the absence of antibiotic use. Also, both strains are susceptible to immune system killing. This model of infection dynamics can be used to search for optimal treatments. *(B)* Schematic representation of the current state of an infection and its treatment. Regular antibiotic is effective against an infection caused by the sensitive strain, but is not effective against an infection with high abundance of resistant pathogens (*B-top*). Here we show that an effective control of the infection can be obtained by initially treating against the resistant strain (antiR condition) [33], [34] and subsequently applying antibiotic treatment (*B-bottom*).

In this paper, we use a mathematical model [42] to quantitatively study antibiotic therapy and the effect of an anti-resistance treatment in a single-host model (Fig. 1A-B). We simulate a case where antibiotic treatment is not effective and show how the application of antiR conditions could provide an effective treatment. Using the model, we are able to estimate for how long (time *t_clear_*) the antiR condition should be applied until antibiotic treatment is again effective. In particular, we show that *t_clear_* depends only on three key parameters: the pathogen division rate, the rate of plasmid loss and the difference in growth rate between sensitive and resistant strains. Also, we use available experimental data to estimate *t_clear_*, providing suggestions on how to manage drug timing in order to clear resistance from a pathogen load. Finally, our single-host model suggests that antibiotic resistance may be attenuated over time. We show that the incorporation of a similar resistance attenuation term into host-population models may change the current perspective on optimal strategies to reduce incidence of antibiotic resistance.

## Methods

### Background

Our current work builds upon a previous model of bacterial infection and immune response, originally proposed to identify strategies to limit the emergence of antimicrobial-resistant bacterial strains [42]. The pathogens are composed of sensitive (represented by the subscript S) and resistant (represented by the subscript *R*) strains. The abundance of pathogens, *B = B_S_+B_R_*, is limited to a carrying capacity *λ·κ* [43]–[45], giving rise to a logistic growth. The growth rate, *λ_S_* or *λ_R_*, is the difference between the division (*δ*) and the mortality (*μ*) rate. The model also considers the effect of the immune system, represented by the number of phagocytes (*P*) and their killing rate (*γ*), and assumes that the populations of pathogens and phagocytes are well mixed. The presence of the immune system effectively translates into a threshold of pathogen abundance, above which an infection starts [46]. The model also assumes that the genes for resistance are carried by mobile genetic elements (referred to in what follows as plasmids, see also Discussion). The resistance-carrying mobile genetic elements can be transferred to a sensitive strain, due to horizontal gene transfer, at a rate *τ*, and be lost during replication, with a probability *ρ* [47]. An illustration of the model and parameters is shown in Figure 1A. Mathematically, the model is described by the following differential equations:
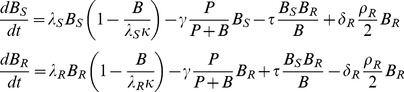
(1)The values for the parameters used in Equation 1 are described in Table S1 in File S1. The different conditions described in this paper (no treatment, antibiotic treatment and antiR) are distinguished by different values of mortality rate and are also described in Table S1 in File S1. Throughout this work, we use a specific fixed value for each parameter and we assume that antibiotic treatment has equal access to each pathogen cell. These assumptions make it easier to understand the model principles and do not affect the conclusions of our analysis. A sensitivity analysis shows that our results are robust to a varying range of parameters (Text S4 in File S1 and Fig. S2).

### Model intuition

The model describes an infection by predicting the dynamical changes in the population of invasive pathogens. If the population is low, the immune system is able to control the infection. When the population is beyond the immune system capacity, the infection needs to be controlled by antibiotic therapy (Fig. S1A,B). However, an infection will not be cured if therapy is interrupted before the pathogen load is sufficiently reduced (Fig. S1B) or if the pathogen population is resistant to antibiotic (Fig. S1D). Also, a time delay in antibiotic application can indicate whether an antibiotic therapy will lead to a successful treatment (Fig. S1C) or not (Fig. S1D). In addition, the relative killing rates of antibiotic and immune system depend on pathogen abundance (see Text S5 in File S1). More details about the model can be found in the original paper [42].

## Results

### Treating against resistance

We used the model of Equation 1 to predict optimal strategies for healing infections that involve strains resistant to a single antibiotic. This is performed by estimating the outcomes of a therapy based on the application of antiR and antibiotic treatment with different time schedules (Fig. *1B*). Antibiotic usage reduces the population of sensitive pathogens while at the same time favoring the resistant ones. If the abundance of the resistant population is too high, antibiotic treatment is ineffective. We explore whether an appropriate timing of the antiR condition [33], [34] could give rise to alternative avenues to combat resistance.

**Figure 2:**
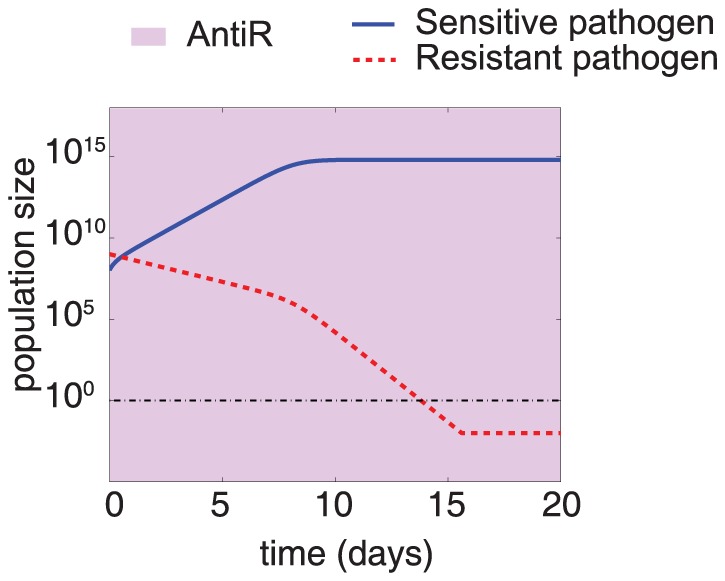
Resistance attenuation is boosted when the population of sensitive pathogens approaches carrying capacity. This figure shows the infection dynamics of both resistant (dashed red line) and sensitive (solid blue line) pathogens under antiR treatment (purple shade). The decrease in the abundance of resistant pathogen is relatively small when the sensitive strain is far from carrying capacity (time t<8 days), but is strengthened when the sensitive population reaches carrying capacity. The initial abundances of sensitive and resistant pathogens are 10^8^ and 10^9^ cells respectively.

**Figure 3:**
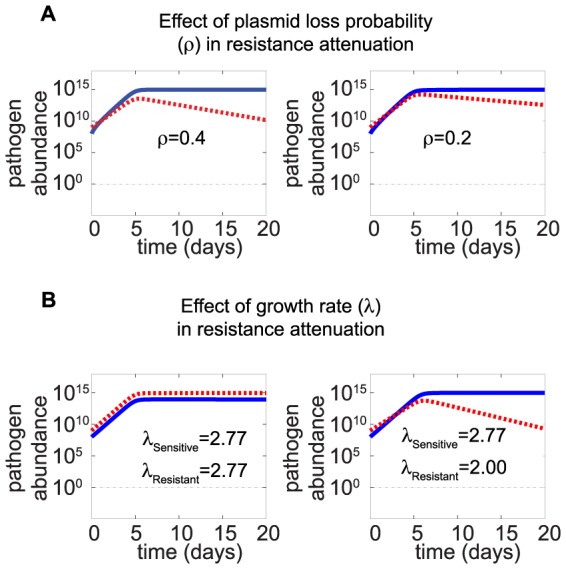
Resistance attenuation occurs in the in the absence of antibiotic treatment when the abundance of sensitive pathogen is saturated. The resistant and sensitive strains have to compete for resources when the bacterial population approaches carrying capacity. This competition reduces the abundance of resistant strains due to the cost of resistance. Under this saturated conditions, both the probability of plasmid loss *(A)* and the growth rate *(B)* affect resistance attenuation. (*A*) The intensity of resistance attenuation increases with the probability of plasmid loss (*ρ*). (*B*) The intensity of resistance attenuation increases with the difference in growth rate between both strains. In this analysis, we set up the probability of resistance loss to be equal to zero to highlight only the effects of growth rate. The left panel shows a case in which both sensitive and resistant strains have the same growth rate. In this case, both strains can coexist with high population abundance. In the right panel, we assume that a plasmid cost reduces resistance growth rate from 2.77 to 2 day^−1^. The abundance of the resistant pathogen decreases over time when the abundance of the sensitive pathogen is saturated. The intensity of resistance attenuation is proportional to the difference in growth rate. Unless otherwise mentioned, all parameters used in this analysis correspond to the default values described in Table S1 in File S1 for no treatment condition. Initial abundances of sensitive and resistant pathogens are 10^8^ and 10^9^ cells respectively.

We studied the effect of an antiR treatment in the infection dynamics and examined how it could help to fight resistant infections. The application of an antiR treatment reduces the abundance of resistant pathogens (Fig. 2). Interestingly, the intensity of this resistance attenuation increases when the abundance of sensitive pathogen is close to the carrying capacity and indicates a change in fitness when both strains have to compete for resources. This phenomenon suggests that competition for resources might also direct resistance attenuation under no treatment conditions. Notably, resource competition has recently been shown, both in terms of mathematical simulations and experimental data, to play a major role in the duration of inflammatory reaction caused by virulent pathogen [48]. We simulated infection dynamics when no treatment is applied to determine the key parameters responsible for resistance attenuation. We observed that the stability of the genes for resistance (represented by the plasmid loss rate) as well as the parameters related to growth rate play a key role in resistance attenuation when the sensitive population is close to carrying capacity (Fig. 3).

**Figure 4:**
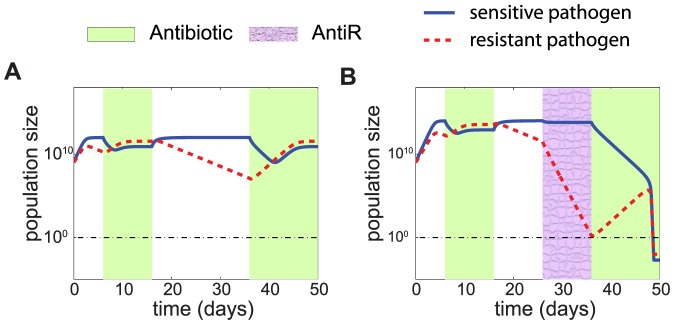
AntiR treatment boosts resistance attenuation and leads to total healing. Both antibiotic suspension (no treatment) and antiR treatment can reduce the abundance of resistant pathogens. However, this reduction is greater under antiR treatment. This figures illustrates the potential advantage of an antiR treatment in fighting a resistant infection. When no treatment is applied, the fraction of resistant population decreases slowly (*A* and *B*, time window between 16 and 36 hours) and it is followed by an ineffective antibiotic treatment. In (*B*), the resistance attenuation is faster due to treatment against resistance (antiR, purple-shaded area), and leads to an effective antibiotic treatment (t>36h). The black dashed horizontal line marks a single cell, i.e. the level below which the infection is healed. The initial abundance of both sensitive and resistant pathogens is 10^9^ cells. Note that the period of antibiotic suspension preceding an antiR treatment is not necessary for an optimal therapy and is shown in this figure only for highlighting the different slopes.

Our goal is to explore the potential of resistance attenuation as an alternative treatment to fight resistant infection. For this purpose, we simulated infection dynamics under different treatment schedules (Fig. 4). Resistance attenuation can be exploited to reduce the population of resistant pathogen to low levels, turning antibiotic therapy effective. The higher the intensity of resistance attenuation, the faster a resistant infection would become sensitive to antibiotic treatment. An antiR condition increases the intensity of resistance attenuation relative to drug suspension and reduces the time it takes for a resistant infection to become susceptible to antibiotic treatment. Figure 4 simulates a case in which antiR treatment leads to an effective treatment that would not be achievable by suspending antibiotic use. This result illustrates the potential of antiR conditions to accelerate resistance attenuation.

Surprisingly, the results of our simulations show that the abundance of sensitive pathogen grows in parallel with the resistant pathogen under antibiotic treatment (Fig. 4B). This phenomenon depends on the simple assumption that the resistance plasmid can be lost: the population of sensitive pathogens could then spontaneously rise to high levels from a high abundance of resistant pathogen.

**Figure 5:**
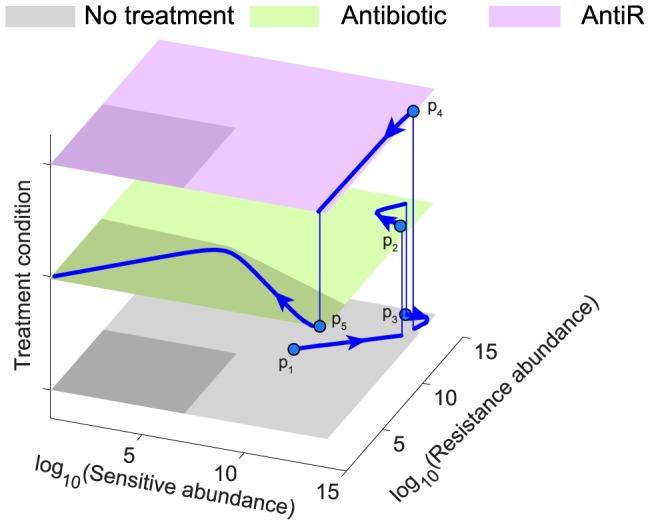
Schematic representation of a phase space shows possible paths for an effective therapy. A phase space shows the growth direction for different size of the resistant and sensitive populations (x and y-axes respectively) upon different types of treatments (different planes on the z-axis). The dark shade in each plane represents the area in which the population of pathogen has negative growth (i.e. infection is under control). In this phase space we display a specific trajectory representative of a therapy that successfully controls resistant pathogens. Each treatment condition is represented as a different plane: no treatment (bottom plane, gray), antibiotic (middle plane, green), antiR (top plane, purple). For the bottom and top planes, the dark shaded area coincides with the population threshold controlled by the immune system. Note that, due to log-scale representation, these areas look like squares. Variations in the parameters for the immune system would cause an extension or contraction of the dark area, without affecting major conclusions from this analysis (see also Text S4 in File S1 and Fig. S2). The use of antibiotic extends the range of control, allowing the cure of infections caused by sensitive pathogens. No single treatment is able to provide cure in all population ranges. However, this can be achieved using multiple treatment therapy. The points (p_1_, …, p_5_) illustrate an effective path (which is the same shown in Fig. *4B*).

The possible outcomes of treatment can be visualized by a schematic phase plane representation (Fig. S3). Note that, according to this schematic representation, no single treatment is effective at treating an infection for all ranges of pathogen populations. However, an effective treatment is possible for any combination of pathogen populations, using a multi-treatment therapy. The infection dynamics for a multi-treatment therapy can be visualized by plotting the phase plane for each individual treatment in a tri-dimensional representation (Figure 5). This representation helps choose the correct strategy to combat infection based on pathogen abundances. It also helps visualize necessary conditions for an effective treatment. In particular, an effective treatment for a full range of pathogen populations requires that the antibiotic treatment is effective even if the abundance of sensitive pathogen is at carrying capacity (Text S4 in File S1 and Fig. S2). A medically relevant outcome of this analysis is that it provides a potential explanation for the prevalence of high-resistant infection in immunosuppressed patients [49], [50] (see Text S4 in File S1).

### Estimating the time to lose resistance

An optimal treatment depends on the precise timing of the application of antibiotic and antiR conditions. If the infection is already sensitive, antibiotic treatment should be used from the beginning of therapy. On the other hand, if the infection is resistant, antiR should be applied first in order to reduce the load of resistant pathogen. When the abundance of resistance is low enough, the infection becomes sensitive and an effective treatment can be achieved after antibiotic application.

The optimal strategy to combat a resistant infection will depend on how the resistant population varies over time. For example, assume that, at a given time *t*, a patient is infected by a given population of resistant pathogen *B_R_(t)*. Under antibiotic treatment, the pathogen carrying the plasmid for resistance will increase in frequency. However, in the absence of antibiotic selection, the cost associated with the plasmid will cause the frequency of the resistant strain to decrease over time (Fig. *4A,B*). What is particularly noteworthy is that under certain conditions (Fig. *4B*) the resistant population can decrease to a level that is low enough, such that the immune system and the antibiotic are able to completely eliminate the pathogens. As shown under no treatment or antiR condition (Fig. 4B) and demonstrated analytically (Text S1, Equation S4 and S5 in File S1), the decrease in abundance of resistant pathogen can be modeled by an exponential function, providing the following phenomenological linear equation:
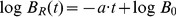
(2)where *a* indicates the rate at which resistance is attenuated (resistance-decaying rate) and *B_0_* the abundance of resistant pathogen at a reference time. The resistance-decaying rate is associated with the cost of resistance and its value increases under antiR conditions.

The expression shown in Equation 2 enables an estimation of the time to lose resistance. To compute this time, it is important to consider the maximum abundance of resistant pathogen that guarantees an effective antibiotic treatment (which we call *h_0_*). We did not find an analytical solution for *h_0_* in terms of the model parameters, but this value can be estimated numerically and visualized in the phase plane representation (Fig. S3B). In addition, a suboptimal estimation of *h_0_* satisfies the requirement for a conservative analysis. In the most conservative scenario, this threshold corresponds to a single resistant pathogen. From this estimate, one can evaluate the time necessary to turn the pathogen population sensitive to antibiotic treatment (Equation 2). In particular, by imposing that the abundance of resistant pathogen should be less than the threshold *h_0_*, in the form *log B_R_<log h_0_*, one obtains:
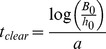
(3)


Note that *t_clear_* is inversely proportional to the resistance-decaying rate. Applying antiR conditions will increase the resistance-decaying rate, consequently decreasing *t_clear_* (Fig. 4).

An analytical approximation derived from the model (Text S1 in File S1) can be used to estimate the resistance-decaying rate and is summarized by the following equation:
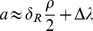
(4)where *Δλ = λ_S_ - λ_R_* is the difference in growth rate of sensitive and resistant strains. *Δλ≈0* when no treatment is applied and it increases under antiR conditions. The parameters *δ_R_* and *ρ* are considered intrinsic to the system [42], but strategies on how to manipulate them might be a topic of future research. Interestingly, the parameters described in Equation 4 coincide with the parameters responsible for resistance attenuation observed under *in vitro* measurement [51].

### Resistance-decaying rate estimated from real data

The applicability of the outlined strategy to fight resistance depends on the ability to realistically estimate the resistance-decaying rate (Equation 4). Experimental measurements of the *ρ* and *δ_R_* parameters can be obtained using the method described in [52], while the parameter *Δλ* can be measured as shown in [53]. In particular, Gill et al. [52] used quantitative real time PCR to measure plasmid counts and a mathematical model to estimate the rate of plasmid loss and *in vivo* growth and death rate, yielding estimates of *ρ* and *δ_R_*. Hegreness et al. [53], conversely, used fluorescence markers to measure differential growth rate between resistant/sensitive strains. Starting from an even population, the intensity of each marker measures the ratio of the abundance of each strain, i.e. 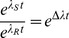.

Empirical data for an antiR condition was obtained from [33]. The authors measured the ratio of doxycycline-sensitive to doxycycline-resistance *Escherichia coli* after 24 hours under control and antiR treatment, which was *1.4* and *150*, respectively. From those values, we obtain *Δλ_ctrl_ = 0.34d^−1^* and *Δλ_antiR_* = *5.01d^−1^*, where the index indicates, respectively, control and antiR conditions.

**Figure 6:**
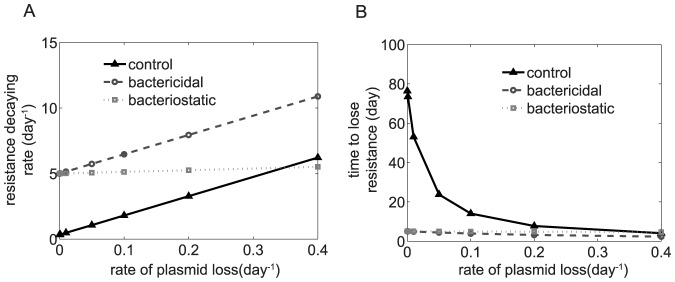
Resistance attenuation is influenced by the nature of antiR treatment and by the plasmid loss rate. The nature of the antiR treatment (whether bactericidal or bacteriostatic, see Text S1 in File S1) and the rate of plasmid loss influence the dynamics of resistance attenuation. We illustrate the resistance decaying rate (*A*) and *t_clear_* (*B*) as a function of the rate of plasmid loss and the nature of treatment. At low rates of plasmid loss (*ρ≈0*), antiR treatment increases the resistance attenuation by a factor ∼15, independently of the nature of antiR treatment. Values are estimated according to data published in [33].

Using the values of *Δλ_ctrl_* and *Δλ_antiR_*, we can compute the resistance-decaying rate (Equation 4) and estimate *t_clear_* (Equation 3) for different values of plasmid loss rate. We estimate that resistance attenuation, measured in terms of *t_clear_*, is boosted up to 15 times under antiR conditions when compared to control conditions (Fig. 6). Moreover, resistance attenuation depends on whether the variation in growth rate is caused by increasing mortality or division rate (see Text S2 in File S1).

### Incorporating resistance attenuation in host-population models

So far, we have explored the concept of resistance attenuation, and its consequences for treatment, based on a single-host model. What would be the implications of introducing the resistance attenuation concept in host-population models of infection? A detailed mapping of the parameters of the single-host model onto those of a host-population model is beyond the scope of the current work. However, we will show here qualitatively how the explicit introduction of resistance attenuation in a host-population model can alter dramatically its predictions, e.g. the effectiveness of drug cycling.

**Figure 7:**
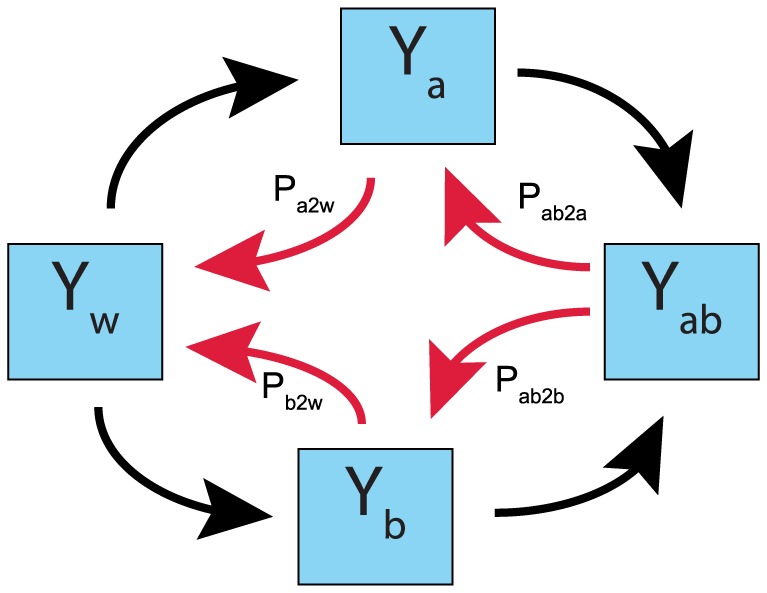
Schematic representation of a host population models that includes the possibility of resistance loss. A modified implementation of a previous host population model [40] under a combination of two drugs *a* and *b* (Equation S6 and S7 in File S1) takes into account the possibility of resistance loss. Hosts can be infected by pathogens of four different types: wild type, a-resistant, b-resistant and a,b-resistant. The numbers of individuals infected are correspondingly represented by variables *y_w_*, *y_a_*, *y_b_*, and *y_a,b_*. The original model [40] considered only the possibility of acquiring resistance (black arrows). In our modified host population model, motivated by our findings in the single host model, we assume that a nonzero resistance-decaying rate can cause loss of resistance (red arrows).

**Figure 8:**
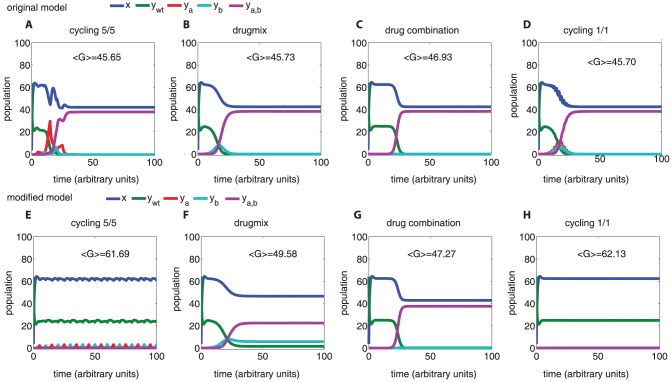
A host population model that takes into account resistance loss leads to different conclusions on the strategies to combat resistance. In the original host population model [40] (see also Fig. 7), drug mixing (panel B) and drug combination (panel C) outperform drug cycling strategies (panel A and D), however, different conclusions can be reached by our modified model (panels E–H). The gain (<*G*>) of therapy is measured by the integral of the curve for uninfected patients (*x*) in each plot. The original model suggests that drug combination provides the best strategy, while the modified model suggest potential gain for cycling. In addition, one can see that cycling periods can be improved to increase gain (compare A *vs D* or E *vs* H). Cycling 5/5: drugs a and b are alternated at every 5 time units; Drugmix 0.5: of patients receive treatment with drug a and 0.5 with drug b; Drug combination: all patients receive both drugs; Cycling 1/1: drugs a and b are alternated at every 1 time unit. Parameters are taken from the original publication [40], with *r_w_* = 0, *r_a_*_ =_ *r_b_*_ = _0.1, *r_ab_*_ = _0.2.

Consider for example the host-population model proposed by Bonhoeffer et al. [40]. In this model, sensitive pathogens can acquire resistance (parameter *s* in Equation S6 in File S1, or Equation 3 in [40]), but there is no parameter explicitly representing the possibility of resistance loss. Rather, in the original model, the cost of resistance is associated with a faster recovering rate. We performed a simulation of the Bonhoeffer et al. model with default parameters and compared it to a modified version that represents transitions from resistant to sensitive strains (see Text S3 in File S1, Fig. S4, Fig. 7). Our analysis shows that adding a term that explicitly refers to resistance attenuation can yield a drastically different conclusion when compared to the original model (Fig. 8), i.e. that cycling is the optimal strategy and that cycling period can be optimized (Fig. 8D and 8H).

## Discussion

Our analysis illustrates a case where a resistant infection could be potentially cured based on the specific timing of two treatments: antibiotic and antiR. An antiR condition can reduce the abundance of a resistant strain by exploiting the cost of resistance. We show that the optimal duration of the antiR administration (*t_clear_*) depends on the resistance-decaying rate, a constant that can be estimated from experimentally measurable parameters [33], [52].

A future potential application of our time-scheduled therapy may be to treat chronic infections, in which resistance turns antibiotic treatment alone unsuccessful [54]–[56]. For example, long-term antibiotic treatment is often ineffective in the treatment of chronic sinusitis [56]. Strategies taking advantage of antiR conditions could be especially useful under conditions in which *t_clear_* is small relative to the timescale of infection progress and a sustained drug suspension or antiR treatment would not threaten the health of the host.

The insight derived from the present analysis is limited by the capacity to effectively implement antiR conditions, and by the assumptions made by the model (Equation 1). For example, an antiR condition obtained through the use of a suppressive interacting drug occurs only at a limited range of drug concentrations, which might not be easily controllable for treatment application. In addition, a pathogen could adapt to an antiR treatment by developing a second resistance. Further important aspects of the way pathogens may cope with antibiotics, such as persistence, compensatory mutations, the development of secondary resistance involving alternative biological mechanisms, as well as a simultaneous application of antibiotic and antiR treatment, are not part of the current investigation, but would be interesting subjects for future expansions.

One of the assumptions of the model described in Figure 1 is that the genes for resistance can be transferred and lost. This assumption is consistent with the integration and excision properties of mobile genetic elements [57]–[60]. De Gelder and colleagues performed experimental measurements that show that plasmid loss due to recombination plays a key role in resistance attenuation [51]; however the rate of transfer and loss of mobile genetic elements is still an under-explored topic [61], [62]. Estimating the extent to which this assumption is true requires specific measurements that are not available in current reports [6], [7], [47], [63]–[67]. Clinical studies usually identify whether an infection is caused by antibiotic-resistant bacteria, but do not measure how the resistance is carried. In addition, most reports on the topic describe resistance to be associated with plasmids. For example, β-lactamases, the most common genes for resistance in *E. coli*, are usually carried by a plasmid [6], [7], [47], [65], [67]. The resistance for quinolones was initially thought to be only caused by serial mutations in the chromosome and to be restricted to vertical transfers. However, 36 years after its introduction, researchers have detected a resistance carrying plasmid that is associated with the rise of high-level quinolone resistance, including multi-drug resistance [7], [63]. The methicillin resistance (*mecA*) in MRSA strains of *S. aureus* is carried in gene cassettes that contain recombinases able to excise and insert them into chromosomal regions [57], [66]. Moreover, most of the resistance to a second class of antibiotics is carried by a plasmid [66]. Resistance-carrying plasmids occur for other classes of antibiotics and organisms and are often the cause for the rise of multi-resistant strains [7], [47].

An important general message emerging from our analysis is that resistance attenuation (which in turns affects *t_clear_*) arises as the population of pathogens approaches its carrying capacity (Fig. 2). This suggests that resource competition is a key component of resistance attenuation, in agreement with previous observations of its role in the selection of resistant strains under antibiotic treatment [68]. A potential implication of this concept is that the population of non-pathogens, by influencing the global carrying capacity [45], [48], may significantly affect the dynamics of pathogens, and should be taken into account for the development of more accurate models.

In addition to exploring in detail the behavior of a single host model under conditions that induce resistance attenuation, we asked ourselves whether resistance attenuation in host population models could affect infection dynamics at an epidemiological level. By introducing a resistance-decaying rate into a previous host population model [40] we found significant changes in the predicted optimal strategy. Most notably while the original model predicts drug mixing or drug combination as the best strategy, our modified model indicates that drug cycling corresponds to the best strategy under otherwise equal conditions. This finding, contingent on further explorations of parameter ranges and assumptions, offers a potential way of reconciling previous contrasting reports of experimentally successful, though theoretically unfavorable, drug cycling therapies [13]–[21], [39]–[42], [69]–[72]. More investigation is necessary to make a mechanist connection between experimentally measurable variables of resistance attenuation and host population models. We believe that a mechanistic understanding of resistance attenuation would be useful in predicting the efficacy of a drug-restriction policy [73], [74].

In the battle against antibiotic resistance, the use of mathematical models is important for transforming the cumulative understanding of the mechanisms for acquisition and loss of resistance [27], [75], [76] into potential strategies to treat infection caused by resistant pathogens. While our work does not suggest an immediate and practical protocol to fight resistant infection, it highlights simple quantitative aspects of resistance attenuation that could eventually translate into novel strategies to fight resistant infections. We envisage that further iterations of empirical and mathematical studies will help understand how specific resistance mechanisms should be incorporated into models to enable improved policies for fighting resistance.

## Supporting Information

Figure S1 Overview of the main properties of the infection dynamics models used in this paper. A treatment is successful when the pathogen population is reduced below the dashed line and is unsuccessful otherwise. The antibiotic treatment is effective when the pathogen abundance has a low fraction of resistance. Panels *(A, B)* illustrate the intuitive effect of different lengths of antibiotic treatment in an infection caused exclusively by the sensitive strain (blue continuous line). The parameters used in this analysis do not affect the qualitative behavior depicted in the original model [42]. A 9 days-long antibiotic treatment (green-shaded region) can reduce infection until the immune system is able to control it (*A*). The same infection is predicted to persist if treatment is interrupted after 6 days (*B*). Panels *C–D* simulated infection dynamics in a mixed population of sensitive (blue continuous line) and resistant (red dashed line) strains. Immediate antibiotic treatment can lead to effective treatment (*C*). However, for the same initial condition shown in (*C*), the abundance of pathogens increases after a 3 days delay under antibiotic use and antibiotic treatment is ineffective (*D*). The initial abundance of sensitive pathogen is 10^8^ for all panels and the initial abundance of resistant pathogen is 10^2^ in panel *C–D* and null for panels *A–B*. The black dashed line in the y-axis highlights when pathogen abundance is equal to a single individual.
(EPS)

Figure S2 Sensitivity analysis of antibiotic treatment effectiveness is illustrated by a schematic phase plane representation. This figure follows the same representation used for the antibiotic plane shown in Figure 5. The dark shaded areas represent regions of the pathogen population that are susceptible to antibiotic treatment, which we refer to as treatable region. For panel (A) and (B), these areas are represented for the reference values, as from Table S1 in File S1. Dashed lines represent the boundary edges of the treatable region for different parameter values. The intersection at the y-axis indicates the population limit for immune-system control. *P_0_* and *μ_0_* indicate reference values, according to Table S1 in File S1. (*A*) Increasing the mortality rate of sensitive strains, *μ_S_*, will expand the treatable region. Notice that the immune-system threshold limits the expansion for the abundance of resistant pathogen. This is visualized by observing that the dark shaded area expands horizontally, but not vertically. Reducing the values of *μ_S_* will shrink the treatable area. At very low mortality rate, it will converge towards the limits for immune-system control. (B) Expansion or contraction of the treatable region as a function of the number of Phagocytes, *P*. *(B–C)* Notice that at low values of *P*, the boundary of the treatable region does not touch the right side edge of the figure. This indicates the treatable region contracts to a level below the carrying capacity. In this case, under antibiotic treatment, the presence of a single resistant pathogen cell will be enough to drive highly abundant sensitive population towards high resistance (*C*, dashed arrow). *(D)* Therapy strategies should consider how drug concentration varies under antibiotic treatment. The treatable region will vary according to *μ_min_* and *μ_max_*, the minimum and maximum values of *μ_S_* during antibiotic treatment. The varying area is represented by vertical hatched area. A conservative strategy should consider the values of *μ_min_* to plan a successful antibiotic treatment.
(EPS)

Figure S3 Schematic representation of infection dynamics depicts success or failure of infection treatment. An infection treatment can typically lead to two possible outcomes: the first is complete healing of the infection, the second persistence of the infection. Each panel shows a schematic representation of infection dynamics under different type of treatment. A successful treatment reduces the total pathogen abundance and directs infection towards the origin (attractor 1). An ineffective treatment is not able to contain infection and pathogen abundance grows towards attractor 2. The dark and light shaded areas represent the regions of pathogen abundance where treatment is effective and ineffective, respectively. (*A*) When no treatment is performed, the immune system is able to control infection of low pathogen abundance. In case of high pathogen abundance, infection ensues and pathogen abundance converges towards a high sensitive population. (*B*) Antibiotic treatment extends the region under which infection can be controlled towards highly sensitive pathogen abundance and moves the position of attractor 2 to a highly resistant infection. Note that the top right corner of the dark shaded area indicates the maximum abundance of resistant pathogen that guarantees an effective antibiotic treatment (*h_0_*, Equation 2). (*C*) An antiR treatment slightly extends the region of pathogen abundance where infection can be controlled towards the population of resistant pathogen. However, in this conservative representation, antiR treatment is not able to control a fully resistant infection. Note that none of the three options of treatment would be successful to treat infection in all range of pathogen abundance. However, our analysis predicts that an effective treatment could be possible for all range of pathogen population in a multi-treatment representation (see figure 5).
(EPS)

Figure S4 Our modified host population model considers a rate of resistance loss that is exponentially proportional to the antibiotic usage. We represent the rate of resistance loss (Equation S7 in File S1) as a function of drug usage. This shape is inspired on the exponential rate of resistance loss suggested by our analysis and also supported by the observed values measured by De Gelder and colleagues [51]. Note that at high-antibiotic usage, this rate is close to null.
(EPS)

File S1 Combined supporting information, containing Table S1, Text S1, Text S2, Text S3, and Text S4.
(PDF)
