# Supplementary material for: Resource Competition May Lead to Effective Treatment of Antibiotic Resistant Infections
Source: PLoS One. 2013 Dec 13;8(12):e80775. doi: 10.1371/journal.pone.0080775 (PMC3862480; doi:10.1371/journal.pone.0080775)
Supplement: File S1 — Combined supporting information, containing Table S1, Text S1, Text S2, Text S3, and Text S4. (PDF) [file pone.0080775.s005.pdf]

Supplementary Information for:

Resource competition may lead to effective treatment of antibiotic resistant infections

Table of Contents

Table S1	Page 2
Text S1	Page 3
Text S2	Page 4
Text S3	Page 5
Text S4	Page 8
Supplementary References	Page 10

SUPPLEMENTARY TABLES

Table S1:

Symbol	Interpretation	Default value	Units
λ	Growth rate	δ-μ	day $^{-1}$
δ	Division rate	2.7726	day $^{-1}$
μ^{a}	Mortality rate	0	day $^{-1}$
κ	$\lambda\kappa$: Carrying capacity	10^{14}/2.7726	-
γ	Killing rate of phagocytes	33.6038	day $^{-1}$
P	Total number of phagocytes	332711	-
τ	Rate plasmid is acquired	10^{-3}	day $^{-1}$
ρ	Probability plasmid is lost	0.4	day $^{-1}$

Description of variables used in the model (Equation 1). Values are chosen according to the original reference [1], unless indicated otherwise in the main text. For the carrying capacity we used a value that is an order of magnitude lower relative to the above reference. This modification is consistent with empirical evidences [2-4] and does not affect the main properties of the model.

[a]mortality rate varied according to treatment conditions (no treatment, antibiotic, antiR) and pathogen strain (sensitive or resistance), with values, respectively to each treatment conditions: μ_S= 0, 4 or 1 for the sensitive strain and μ_R= 0, 0 or 3 for the resistant strain.

SUPPLEMENTARY TEXT

Text S1. Analytical derivation of the resistance-decaying rate

The parameters to compute the resistant decaying rate (*a* in Equation 2, Equation 3, also illustrated in Fig. 4) can be mathematically derived from Equation 1. First, let's consider the part related to logistic growth. If the pathogen population is near saturation level, i.e., $B(t)=B_S(t)+B_R(t) = \lambda_S\kappa$, then the corresponding parts in Equations 1 reduce to:

$$\frac{dB_S}{dt}\Big|_{\text{logistic}} = \lambda_S B_S(t) - \frac{B(t)}{\kappa} B_S(t) = 0$$
$$\frac{dB_R}{dt}\Big|_{\text{logistic}} = \lambda_S B_R(t) - \frac{B(t)}{\kappa} B_R(t) - \Delta\lambda B_R(t) = -\Delta\lambda B_R(t) \qquad \text{[S1]}$$

where $\Delta\lambda = \lambda_S - \lambda_R$.

At this regime, using the known parameter, we have $B(t) \approx 10^{14}$ and $\gamma P < 10^8$. In turn, these values imply that immune system response will be close to 0, i.e.

$$\gamma \frac{P}{P+B(t)} B_S(t) < 10^{-6} B_S(t)$$
$$\gamma \frac{P}{P+B(t)} B_R(t) < 10^{-6} B_R(t) \qquad \text{[S2]}$$

Under these conditions, the sensitive population will be close to the total population, i.e., $B_S(t) \approx B(t)$. Thus, variation in population can be approximated by:

$$\frac{dB_S}{dt} = -\tau \frac{B_S(t)B_R(t)}{B(t)} + \delta_R \frac{\rho}{2} B_R(t) \approx B_R(t)(-\tau + \delta_R \frac{\rho}{2})$$
$$\frac{dB_R}{dt} = \tau \frac{B_S(t)B_R(t)}{B(t)} - \delta_R \frac{\rho}{2} B_R(t) - \Delta\lambda B_R(t) \approx B_R(t)(\tau - \delta_R \frac{\rho}{2} - \Delta\lambda) \qquad \text{[S3]}$$

Since τ is much smaller than $\delta_R \frac{\rho}{2}$ and $\Delta\lambda$, and $\frac{B_R(t)}{B_S(t)} \approx 0$, we can simplify Equation S3 and finally get:

$$\frac{d}{dt}\log(B_S(t)) \approx \frac{B_R(t)}{B_S(t)}(\delta_R \frac{\rho}{2}) \approx 0$$
$$\frac{d}{dt}\log(B_R(t))' \approx -\delta_R \frac{\rho}{2} - \Delta\lambda \qquad [S4]$$

The resistance decaying rate, i.e. the coefficient *a* of Equation 2, can be taken from Equation S4 and it will be equal to

$$a \approx \delta_R \frac{\rho}{2} + \Delta\lambda \qquad [S5]$$

Equation S5 is also represented in the main text, labeled as equation 4. It is repeated here to provide better comprehension of the text. The coefficient *a* can be substituted in Equation 3 to estimate t_{clear}. The quality of this analytical approximation was compared to measurements from the simulations shown in Fig. 4, with estimated value at least 96% of the exact value.

Text S2. The mechanism of action of antiR treatment affects the resistance-decaying rate.

The estimated value for the resistance decaying rate *a* (Equation S5), and therefore of the time to lose resistance t_{clear} (see Equation 3), depends on whether antiR treatment affects the growth by changing the division or the mortality rate, i.e. on whether a drug is bacteriostatic or bactericidal respectively. A purely bactericidal mechanism affects growth rate by increasing mortality rate (μ_R), while the effect of a purely bacteriostatic treatment reduces division rate (δ_R). Hence the dependence on the

type of antibiotic can be seen by a careful analysis of Equation S5, which can be rewritten more explicitly as $a \approx \delta_R\rho/2 + \lambda_S - (\delta_R-\mu_R)$.

In particular, at low enough rates of plasmid loss (ρ), the first term in equation S5 becomes negligible, and the resistance decaying rate will depend only on the net difference between division and mortality rates (i.e. the directly measurable growth rate), irrespective of whether the antibiotic is bacteriostatic or bactericidal. On the contrary, at high rates of plasmid loss, the division rate of resistant bacteria (δ_R) has to be explicitly taken into account through the first term of Equation S5.

The method developed in [5] can measure $\Delta\lambda$, i.e. the growth rate, under different conditions. Since δ_R is relevant to compute the resistance-decaying rate, it is important to state if the reduction on the value of λ is caused by increasing μ (bactericidal) or by decreasing δ (bacteriostatic). A drug might not be purely bacteriostatic or purely bactericidal. In such cases, resistance-decaying rate would assume a value between the pure bacteriostatical and pure bactericidal cases.

Experimental measurement of the rate of plasmid loss (ρ) and *in vivo* division rate (δ_R) can be obtained according to the method developed by Gill et al. [6]. In this method, ρ is measured by quantitative real time PCR. The value of δ_R can be estimated from a mathematical model that considers *in vivo* measurement of growth rate and plasmid loss.

Our model predicts that at high rates of plasmid loss, purely bactericidal treatments are more effective in combating resistance. However, the nature of treatment mechanism is not relevant if the plasmid loss is a rare event. Details about it are shown in the main text (Fig. 1).

Text S3. Modified Bonhoeffer et al. model for dynamics of infection in a human host population

Bonhoeffer et al. proposed a mathematical model to describe the benefit of

different drug therapies against resistant bacteria[7]. The model describes the dynamics of infection in a host population. In their model, individuals can be contaminated by four different bacteria strains, characterized based on their sensitivity or resistance to two drugs (a and b). The following equation describes our modified version of the model (where we highlight in red the parts we modified) (see Fig. 7, main text):

$$\frac{dx}{dt} = \lambda - d\cdot x - b(\sum_{i\in(w,a,b,ab)} y_i) + (\sum_{i\in(w,a,b,ab)} r_i y_i) + \\ + h(1-q) f_{ab} y_w + h(1-s)(f_a + f_b) y_w + \\ + f_a y_b + f_b y_a + f_{ab}(y_a + y_b)$$

$$\begin{aligned}
\frac{dy_w}{dt} &= (bx - c - r_w - h(f_a + f_b + f_{ab}))y_w + \alpha(y_a \cdot p_{a2w} + y_b \cdot p_{b2w}) \\
\frac{dy_a}{dt} &= (bx - c - r_a - h(f_b + f_{ab}))y_a + hsf_a y_w + \alpha(-y_a \cdot p_{a2w} + y_{ab} \cdot p_{ab2a}) \\
\frac{dy_b}{dt} &= (bx - c - r_b - h(f_a + f_{ab}))y_b + hsf_b y_w + \alpha(-y_b \cdot p_{b2w} + y_{ab} \cdot p_{ab2b}) \\
\frac{dy_{ab}}{dt} &= (bx - c - r_{ab})y_{ab} + hs(f_{ab}(y_a + y_b) + f_a y_b + f_b y_a) + qhf_{ab} y_w - \alpha \cdot y_{ab}(p_{ab2a} + p_{ab2b})
\end{aligned} \quad [S6]$$

The variables for the original model are:

x : uninfected population.

w, a, b, ab: indexes for wild type, a-resistant, b-resistant and ab resistant strains respectively.

y_i : abundance of hosts infected with pathogen *i in (wt, a, b, ab*).

λ : rate of uninfected host entering the system.

d : death rate of uninfected population.

b : transmission rate.

r_i : recovery rate from infection of type *i in (wt, a, b, ab*).

h : maximum recovery rate under antibiotic treatment.

f_i : fraction of patients treated with antibiotic *i in (a, b, ab*).

c : death rate of infected patients.

s : fraction of hosts that becomes resistant when treated with single drug.

q : fraction of hosts, which becomes resistant when treated with both drugs simultaneously

The new model considers specific distinct paths to lose resistance, whose rates are represented by the terms p_{a2w}, p_{b2w}, p_{ab2a} *and* p_{ab2b} (see Fig. 7), hence adding reversibility to the paths for acquiring resistance in the original model. The single-host analysis we presented in the main text suggests that, in the absence of antibiotic use, the resistance to antibiotic is lost according to the resistance-decaying rate (Equation 4). Here, we explore the implications of this individual-host resistance-decaying rate to the dynamics of infections at the population level. The resistance decaying rate, as derived in the main text, suggests that resistance is lost exponentially in the pathogen, thus we assume that the new rates of population-level loss of resistance p_{a2w}, p_{b2w}, p_{ab2a} and p_{ab2b} are exponentially decreasing functions of the corresponding fractions of infected hosts as defined in the following equation:

$$\begin{aligned} p_{a2w} &= e^{-k(f_a+f_{ab})} \\ p_{b2w} &= e^{-k(f_b+f_{ab})} \\ p_{ab2a} &= e^{-k(f_a+f_{ab})} \\ p_{ab2b} &= e^{-k(f_b+f_{ab})} \end{aligned} \qquad [S7]$$

For the purpose of this paper, we assumed k=5 and α=1 (in equation S6) to illustrate the qualitative effect of the modified Bonhoeffer model. Note that the rate of resistance loss is close to 0 when the fraction of patients using the corresponding antibiotic approaches 1 (Figure S4).

Using this modified model, we studied the effect of different drug treatment strategies (cycling, mixing, combination) on a population of hosts, and compared results with the original Bonhoeffer population model. The original model indicates that drug cycling favors double resistant infection (Fig. 8A). However, a different conclusion can be achieved when the rate of plasmid loss is considered (Fig. 8E). Also, the modified model shows that drug cycling can outperform drug mixing and drug combination (Fig.

8A-C and Fig. 8D-F). In addition, in support to the conclusions illustrated in Fig. 7, we show that better control of resistance can be achieved by improving cycling period.

Text S4. Sensitivity analysis

The qualitative results described in the main text are robust to a wide range of parameters. In this section, we show how the parameters for growth rate and for the immune system affect the outcome of our analysis. Two representative parameters, the sensitive strain mortality rate, μ_S, and the number of phagocyte cells, *P,* are chosen for this purpose. Varying the mortality rate provides a similar behavior for what would be observed by varying the division rate. A similar association links the effects of varying the number of phagocytes to varying the phagocyte-killing rate. The standard values that define the antibiotic conditions, as represented in table S1, are shown as a reference for the sensitivity analysis comparison.

Results of this sensitivity analysis are shown in Figure *S2*. One main result of this analysis is that tuning of the parameters mentioned above (μ_S, and *P*) affects the boundaries of the pathogen abundance region in which infection can be successfully treated (Fig. 5 and S2). Increasing the mortality rate of the sensitive strain (μ_S) increases the region of resistant pathogen abundance that can be contained in a successful treatment. Notice that this abundance is limited by the immune-system capacity (*Fig. S2A*). Conversely, decreasing the mortality rate will shrink the area of this treatable region. At low mortality rate, the presence of a single resistant cell is, in principle, enough to cause antibiotic treatment not to be effective for an infection saturated with sensitivity strain (*Fig. S2C,* arrow path). At very low value, the efficacy of an antibiotic treatment reduces to the immune-system control threshold.

Changing the number of phagocytes (*P*) affects the top edge boundaries for a successful treatment, and thus affects the threshold for the region of immune-system control and also the maximum abundance of resistant pathogen that can be contained in a successful treatment (*Fig. S2B*). By increasing (decreasing) the number of

phagocytes, the population area for an effective treatment tends to expand (shrink). This sensitivity analysis also provides a potential explanation for the observed increased cases of resistance in immunosuppressed patients [8,9]. In particular, one can see that the boundary for the pathogen population representing a treatable region is reduced for immunosuppressed patients. Below a certain threshold of *P* (and for a pathogen population near the carrying capacity) the antibiotic treatable area becomes null. Thus, the presence of even a single resistant pathogen cell would be enough to drive a saturated population of sensitive strains towards a high-resistant infection (*Fig. S2C,* arrow path).

As shown in *Figure S2D*, this sensitivity analysis can also be used to estimate how pharmacokinetics and pharmacodynamics (e.g. time-dependent drug concentration in the patient's body after a single dose of antibiotic) could affect the global treatment plan.

Text S5. Antibiotic and immune-system killing rate efficiency.

The results of our simulations indicates that drugs are more effective than the immune system at killing the pathogen for a large load of pathogen and the immune system is more effective in case of small pathogen population. The short explanation for this is that the killing rate caused by antibiotics is proportional to the total number of cells and the killing rate of the immune-system saturates according to phagocyte abundance and efficiency.

For illustration purpose, we compare the killing rate caused by the immune-system and antibiotic treatment with numbers, let's say that antibiotic kills at a rate of 10% of the total cells and that the immune-system saturates at an efficiency of 10^6 cells, both relative to the same unit of time. Antibiotic treatment would be much more effective to treat a pathogen load of 10^{10} cells and would kill 10^9 cells per unit of time while the immune system at its best efficiency would kill only 10^5 . However, the antibiotic efficiency would be weak for a case of low pathogen abundance, say 10^4. The antibiotic killing rate would be 10^3 while the immune-system would be able to kill all cells at its best efficiency.

Supp. References

1. D'Agata E, Dupont-Rouzeyrol M, Magal P, Olivier D, Ruan S (2008) The Impact of Different Antibiotic Regimens on the Emergence of Antimicrobial-Resistant Bacteria. PLoS ONE 3: e4036.
2. Dall'Antonia M, Coen PG, Wilks M, Whiley A, Millar M (2005) Competition between methicillin-sensitive and -resistant Staphylococcus aureus in the anterior nares. The Journal of hospital infection 61: 62-67.
3. Lipsitch M, Dykes JK, Johnson SE, Ades EW, King J, et al. (2000) Competition among Streptococcus pneumoniae for intranasal colonization in a mouse model. Vaccine 18: 2895-2901.
4. Smith VH, Holt RD (1996) Resource competition and within-host disease dynamics. Trends in ecology & evolution 11: 386-389.
5. Hegreness M, Shoresh N, Hartl D, Kishony R (2006) An Equivalence Principle for the Incorporation of Favorable Mutations in Asexual Populations. Science 311: 1615-1617.
6. Gill W, Harik N, Whiddon M, Liao R, Mittler J, et al. (2009) A replication clock for Mycobacterium tuberculosis. Nature medicine 15: 211-214.
7. Bonhoeffer S, Lipsitch M, Levin B (1997) Evaluating treatment protocols to prevent antibiotic resistance. Proc Natl Acad Sci USA 94: 12106-12111.
8. Jiang J-R, Yen S-Y, Wang J-Y (2011) Increased prevalence of primary drug-resistant pulmonary tuberculosis in immunocompromised patients. Respirology (Carlton, Vic) 16: 308-313.
9. Osman AS, Jennings FW, Holmes PH (1992) The rapid development of drug-resistance by Trypanosoma evansi in immunosuppressed mice. Acta Tropica 50: 249-257.
10. Chait R, Craney A, Kishony R (2007) Antibiotic interactions that select against resistance. Nature 446: 668-671.
